# Nanoscale Thin-Film Flexible Organic Field-Effect Transistors with Triple PMMA/SiO_2_/ZnO Gate Insulator Layers

**DOI:** 10.3390/mi17030382

**Published:** 2026-03-21

**Authors:** Sundes Fakher, Furat AI-Saymari, Mohammed Mabrook, Hameed Al-Attar

**Affiliations:** 1 Department of Physics, College of Education for Pure Sciences, University of Basrah, Basrah, Iraq; furat.alsaymari@uobasrah.edu.iq (F.A.-S.); halattar53@gmail.com (H.A.-A.); 2 School of Electronic Engineering, Bangor University, Dean Street, Bangor LL57 1UT, UK; 3 Department of Physics, University of Durham, Durham DH1 3LE, UK

**Keywords:** flexible device, nanoscale, organic field-effect transistor, triple insulator layers

## Abstract

Organic field-effect transistors (OFETs) incorporating a triple insulating layer of polymethyl methacrylate (PMMA), silicon dioxide (SiO_2_), and zinc oxide (ZnO) were successfully fabricated on glass and on flexible PET substrates. The insulating layers significantly enhanced device performance, with the OFETs achieving field-effect mobility (*µ*) values more than twice as high as those reported in the literature. Specifically, mobility values of ~6.75 cm^2^/V·s were recorded on glass, ~7.14 cm^2^/V·s on flexible substrates before bending, and ~6.88 cm^2^/V·s on flexible substrates after bending. Threshold voltages (*V_th_*) of −7 V and −9 V were estimated for the flexible OFETs before and after bending, respectively, along with a high on/off current ratio, exceeding 10^3^ for all devices. Minimal hysteresis in the transfer and output characteristics indicated excellent, trap-free interaction between the insulating layers and the pentacene. The high dielectric constant of the PMMA/SiO_2_/ZnO triple insulating layers was identified as a critical factor driving the exceptional performance, stability, and low hysteresis of the OFETs. These results underscore the pivotal role of advanced insulating layers in optimizing OFET performance and durability.

## 1. Introduction

Flexible electronics refer to a broad class of thin-film electronic devices that combine high electrical performance with the ability to be bent, stretched, folded, or twisted into unconventional shapes. Their mechanical compliance allows seamless integration with biological systems, enabling numerous human-friendly applications such as smart prosthetics, electronic skins [1], and wearable systems for health and activity monitoring [2,3]. A central challenge in flexible organic electronics is balancing mechanical flexibility with optimal electronic properties [4]. Owing to their intrinsic flexibility and low-temperature processability, organic materials can be deposited onto bendable substrates without suffering significant electrical degradation [5,6]. This capability has accelerated progress in next-generation organic devices based on small molecules and polymers, attracting strong interest from both academic and industrial communities [7,8]. As a result, organic materials have been employed in a wide range of flexible electronic technologies, including organic solar cells [9,10], light-emitting displays [11,12], sensors [9,13,14], memory devices [15,16], organic integrated circuits [3,17], and organic field-effect transistors (OFETs) [18,19,20].

Recent developments in nanoscale organic electronics increasingly focus on replacing traditional rigid substrates, such as glass, with flexible alternatives like metallic foils and polymeric plastics [9,13,17]. A variety of soft substrates—including polyethylene-2,6-naphthalate (PEN), poly(ethylene terephthalate) (PET), parylene, textiles, paper, and elastomers such as polyimide (PI) and poly(dimethylsiloxane) (PDMS)—have been used for flexible device fabrication. For practical deployment, however, the stability and operational lifetime of flexible devices under both normal and extreme environmental conditions remain essential considerations.

Among these substrates, PET stands out due to its low cost, light weight, good moisture resistance, and good thermal stability [21]. As a polyester material, PET may be either rigid or flexible depending on thickness. It possesses a dielectric strength of ~60 kV/mm, a Young’s modulus of 2800–3100 MPa, and a melting point near 250 °C [22]. PET also offers high optical transmission (~85%) and mechanical robustness under bending, making it an attractive candidate for flexible displays and printed electronics [13,23]. Typical printed-electronics applications employ PET films with thicknesses between 20 and 100 μm [13,24].

Organic field-effect transistors (OFETs) are key building blocks for organic electronic systems, and they can serve as amplifiers, switches, drivers, memory elements, and sensors [19,25]. Their compatibility with low-temperature processing enables fabrication directly onto a wide range of flexible substrates [26]. Although OFET performance has advanced substantially—supporting flexible, low-cost, and large-area electronics—several challenges persist, including operational instability under ambient conditions, contact resistance issues, and the limited air stability of many n-type materials. Recent studies have shown that molecular design strategies such as fluorination can improve ambient stability by suppressing moisture uptake [27]. Donor–acceptor polymer semiconductors have also demonstrated record electron mobilities (~1.3 cm^2^/V·s) and minimal threshold-voltage shifts under prolonged bias stress, illustrating that both high performance and stability are achievable [28]. OFET-based sensors similarly benefit from mechanical flexibility and tunable interfacial chemistry, yet their performance is still constrained by low mobility, limited environmental stability, and slow response and recovery dynamics [29]. Improvements in gate dielectrics—such as polymeric layers incorporating internal dipoles—have proven effective in reducing trap densities, lowering operating voltages, and enhancing stability [30].

Dielectric engineering has therefore become central to OFET optimization. Devices incorporating double or multi-layer gate insulators have been widely investigated [31,32], with many reports demonstrating that high-k dielectric layers can reduce threshold voltage and enhance field-effect mobility in flexible OFETs [31,33,34]. Moreover, appropriate dielectric selection strongly influences the growth and crystallinity of the organic semiconductor, enabling larger grain sizes and improved molecular ordering [35,36].

In this study, we examine the effect of introducing a triple-layer gate dielectric stack on the performance of pentacene-based OFETs fabricated on both flexible PET and rigid glass substrates. The mixed dielectric structure significantly influences charge-carrier mobility and enables consistent device performance even under un-encapsulated conditions. To evaluate interfacial behavior at the pentacene/dielectric and dielectric/dielectric boundaries, current–voltage characteristics were recorded in both forward and reverse sweeps. The resulting transfer curves exhibit minimal hysteresis, demonstrating the stability and reliability of the fabricated flexible OFETs.

## 2. Experimental Details

**Figure 1:**
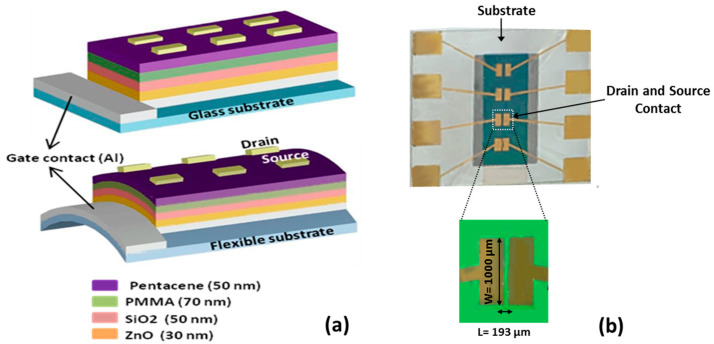
(**a**) Schematic diagrams of the pentacene-based OTFTs on glass and flexible substrate; (**b**) a microscopic image of the top view of the fabricated pentacene-based OTFTs.

The materials used in this work—pentacene, anisole, and PMMA (with a molecular weight of 93,000)—were provided by Sigma-Aldrich, Gillingham, UK. All devices were made at room temperature. Figure 1 shows the structure of the OFETs (Al/ZnO/SiO_2_/PMMA/pentacene/Au) built on both glass and flexible plastic (PET) substrates (Figure 1a) along with a microscopic image of the top view of fabricated device (Figure 1b).

To build the devices, a 50 nm thick aluminum (Al) layer was first deposited onto the substrates using thermal evaporation through a shadow mask to form the gate electrode. Next, a 30 nm layer of ZnO was added as the first insulating layer, forming a defect-free, column-like nanocrystalline structure. A second insulating layer, 50 nm of SiO_2_, was then added using glow-discharge sputtering in an oxygen environment.

The third insulating layer, PMMA, was applied by spin coating a 10% PMMA–anisole solution at 5000 rpm for one minute, forming a 70 nm layer. This layer was then heated at 120 °C for one hour to solidify. After that, a 50 nm thick pentacene layer, serving as the organic semiconductor, was deposited by thermal evaporation at a slow rate of 0.03 nm/s using another shadow mask. Finally, a 50 nm thick gold (Au) source and drain electrodes were deposited on top using thermal evaporation through a mask.

The electrical performance of the devices was tested using a, source-meter, Keithley Instruments, Ohiu, USA, measuring the current–voltage (*I*–*V*) characteristics in both directions. These tests were done at room temperature (about 21 ± 2 °C), and then the devices were stored under vacuum to prevent degradation from air exposure.

## 3. Results and Discussion

**Figure 2:**
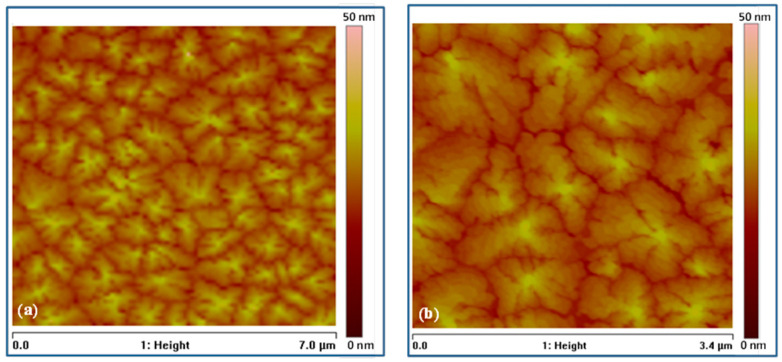
AFM images of the pentacene thin film grown on PMMA/SiO_2_/ZnO triple-gate insulator layers with scan area sizes of (**a**) 7.0 µm and (**b**) 3.4 µm.

Figure 2 shows atomic force microscopy (AFM) images of pentacene thin film grown on PMMA/SiO_2_/ZnO triple-gate insulating layers of 150 nm thickness. The average grain size of the 50 nm pentacene film was about 1.94 μm^2^, with an average roughness of Ra = 2.86 nm. The topography of the sample surface shows that the pentacene is composed of large-size grains with significant terraces, indicating that the crystallinity of the pentacene layer is very high [35,36]. The substantial grain sizes demonstrated significance for enhanced mobility of pentacene, facilitating increased current when used as the active layer in organic FETs. Comparison of these results with our previous result, obtained when a single-gate insulating layer of PMMA was used [37], indicates that a pronounced enhancement of crystallinity was achieved when triple-gate insulating layers were used.

It was observed that immediately depositing gold (Au) source and drain contacts onto a freshly prepared pentacene layer resulted in poor device performance. Better-performing devices were achieved when the pentacene layer was kept under vacuum for several days before the Au contacts were added. This improvement is likely due to the need for the pentacene layer to fully dry and stabilize, possibly through a slow annealing process, before gold deposition.

When 50 nm thick Au contacts were deposited on a 50 nm pentacene layer that had been left to stabilize, the resulting film showed a uniform, pinhole-free, and crack-free surface. In contrast, Au contacts deposited immediately after pentacene evaporation showed surface defects. These differences in Au–pentacene film morphology highlight the importance of allowing the pentacene layer sufficient time to stabilize before metal contact deposition.

**Figure 3:**
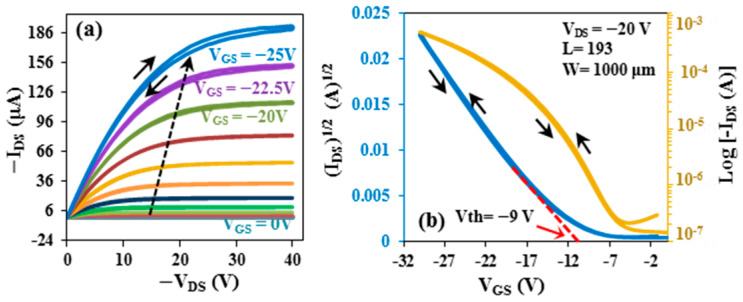
The (**a**) output and (**b**) transfer characteristics of the OTFT on glass substrate.

In this study, the same fabrication procedure was applied both to flexible OFETs and to those on glass substrates. The thicknesses of the pentacene and gold layers, as well as the deposition methods, were kept identical to ensure consistency across all devices. The plot of typical output characteristics of a p-channel OFET on glass substrate represents the dependence of drain–source current (*I_DS_*) on the drain–source voltage (*V_DS_*) with regard to the gate bias from 0 to −25 V with 2.5 V steps, as shown in Figure 3a. Drain–source current increases as gate bias voltage increases, exhibiting typical p-type OFET behavior with a clear saturation trend.

Drain–source current (*I_DS_*) in the saturation regime is related to gate–source voltage (*V_GS_*) by the following formula [38,39]:

$$I_{DS}=\frac{\mu WC_{i}}{2L}{\left(V_{GS}-V_{th}\right)}^{2}$$

where *W* is the channel width, *L* is the channel length, *μ* is the field-effect mobility, *C_i_* is the insulator capacitance per unit area, and *V_th_* is the threshold voltage. In this work, the channel width (*W*) is 1000 μm and the channel length (*L*) is 193 μm. By measuring the equivalent metal–insulator–metal (MIM) device fabricated on glass and flexible substrates, the dielectric capacitance was determined. The corresponding capacitances of ZnO, SiO_2_, and PMMA were almost 248, 69, and 35 nF/cm^2^, respectively. The slope of the (*I_DS_*)^1/2^ versus *V_GS_* plot at *V_DS_* = −20 V is demonstrated in Figure 3b. The transfer characteristic was also evaluated by plotting log (*I_DS_*) versus *V_GS_* (as shown in Figure 3b). It is clear from Figure 3b that the parameters of the OFET on the glass substrate had significant values, where the on–off ratio was determined to be 3.70 × 10^3^ and the field-effect mobility, *µ*, to be about 6.75 cm^2^/V.s, with a threshold voltage, *V_th_*, of −9 V.

**Figure 4:**
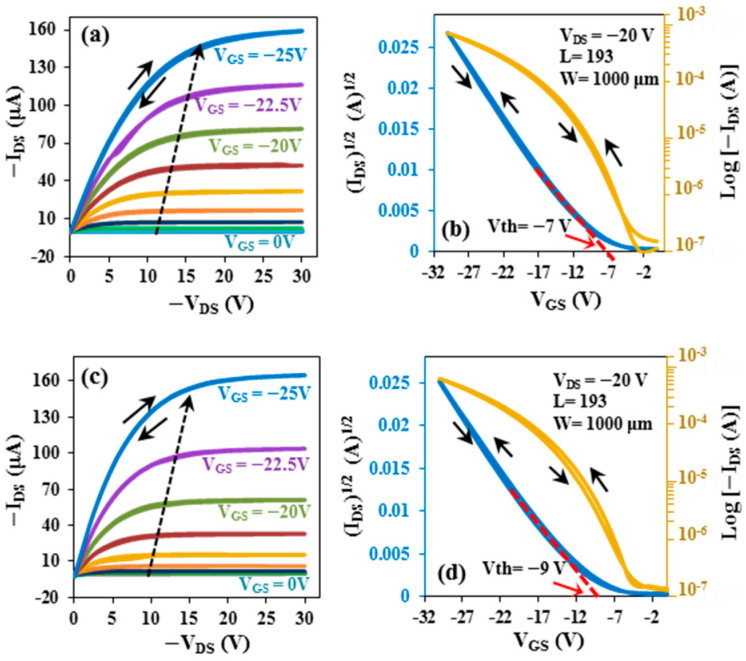
Output (**a**) and transfer (**b**) characteristics of OTFT on flexible PET substrate before bending. Output (**c**,**d**) transfer characteristics after bending.

To evaluate the reproducibility and reliability of the fabricated OFET devices, a total of 60 flexible devices were fabricated under identical processing conditions. Among them, 52 devices exhibited stable electrical characteristics and were included in the statistical analysis of the key electrical parameters. The electrical performance reported in the manuscript corresponds to representative devices, while the statistical distribution and variation of the measured parameters are summarized in the Supplementary Information. Figure 4 shows the *I*-*V* output characteristics (Figure 4a) and transfer characteristics (Figure 4b) for OFETs on flexible substrates before bending, while Figure 4c,d show the *I*-*V* output and transfer characteristics, respectively, of OFETs after bending. The *I*-*V* characteristics, expressing drain–source current as a function of drain–source voltage, were measured over the gate bias range with steps of 2.5 V from 0 to −25 V (see Figure 4a,c), and showed that the current increased as the gate voltage increased. The results also indicated that typical p-type OFET behavior with an excellent saturation trend was observed.

The transfer characteristics of the OFETs were measured at *V_DS_* = −20 V, as shown in Figure 4b,d. From these figures, OFETs before bending were found to exhibit an on–off ratio of 7.10 × 10^3^, a field-effect mobility, *µ*, of about 7.14 cm^2^/V.s, and a threshold voltage *V_th_* of −7 V. After bending, the results for OFETs showed that the on–off ratio was 4.40 × 10^3^ and field-effect mobility was about 6.88 cm^2^/V.s, with a −9 V threshold voltage. Furthermore, no noticeable leakage current was observed in these devices. The extracted mobilities represent effective field-effect mobilities and may include minor contributions from contact resistance. Because all devices were fabricated under identical conditions, the comparative mobility analysis remains valid. The mechanical robustness of the flexible OFET devices was evaluated by performing cyclic bending tests. The devices were bent to a bending radius of 5 mm, imposed by hand with a gap guide. Each device was subjected to approximately ten bending cycles to investigate the effect of mechanical strain on device performance. After cyclic bending tests, 48 of 52 devices retained stable electrical performance, indicating good mechanical robustness of the flexible OFET devices after bending (more information in the Supplementary Information). Considering that using a very thin substrate is the first requirement for producing transistors that could be bent to a small radius [24], the transistor layers were applied to 100-micron-thick polyethylene terephthalate (PET) sheets. The significant parameters of the flexible OFETs before and after bending demonstrated high performance with reasonably high mobility, clear evidence of improved device behavior.

The results reported in this work show the highest mobility recorded to date, compared to flexible OFETs previously reported in the literature which were based on pentacene with different insulating layers [31,36,37,38,39,40,41,42,43]. This is attributed to the fact that the field-effect mobility and threshold voltage of the flexible OFETs could be enhanced using high-dielectric (high-k) insulating layers, where a large grain size and a high crystallinity are achieved [31,33,34,35,36]. The main electrical and mechanical properties of our flexible OFETs compared to those of devices previously reported in the literature are listed in Table 1.

The results presented in Table 2 clearly demonstrate the impact of different dielectric layer configurations on the performance of pentacene-based OFETs. Earlier reports employing single polymeric dielectrics such as PMMA or PVP, with dielectric thicknesses in the range of 300–350 nm, typically exhibited moderate mobilities between 0.27 and 1.32 cm^2^/V.s and relatively high threshold voltages (−7 to −16 V) [37,45,46]. Similarly, devices incorporating single AlOx or binary dielectric combinations (AlOx/ODPA or AlOx/PMMA) showed even lower mobilities, ranging from 4.0 × 10^−4^ to 0.1 cm^2^/V.s, with threshold voltages around −2.8 to −12.5 V [47]. These results suggest that while conventional single or double dielectric layers can ensure device operation, they often suffer from either poor charge transport or unfavorable threshold voltage characteristics.

In contrast, the present work employing a triple-layer dielectric structure (ZnO/SiO_2_/PMMA) demonstrates a significant improvement in device performance. On glass substrates, the mobility increased to 6.75 cm^2^/V.s with a threshold voltage of −9 V, while on flexible substrates, mobilities as high as 5.88–7.14 cm^2^/V.s were obtained with a threshold voltage of −7 V. These values represent a substantial enhancement compared with devices utilizing only one or two dielectric layers, indicating that the optimized triple-layer configuration effectively combines the advantages of inorganic and polymeric dielectrics. This approach not only improves charge carrier mobility but also stabilizes threshold voltage, highlighting the potential of multi-layer dielectric engineering for high-performance and flexible OFET applications.

The significant performance of flexible OFETs is due to the three layers of the insulating material, especially the ZnO and SiO_2_ layers. The pure ZnO layer with a thickness of 30 nm (the first insulator layer on the Al contact) played an important role in avoiding damage to the gate layer during bending, exhibiting an enhancement in the OFET results. The silicon layer between the ZnO and PMMA layers improves the behavior of the device in terms of its flexibility, and increases its effectiveness. As for the last layer of the insulating material, PMMA, its presence directly below the active layer, pentacene, enhances the performance of the transistor device. This enhancement was also achieved and discussed in previous works [37,48]. In comparison with PMMA, ZnO exhibits a higher dielectric constant. This could contribute to a large gate capacitance and thus reduce the operating voltage. ZnO can also be used to reduce the interface trap density within the semiconductor layer, leading to improvement in the charge transport process [49,50]. SiO_2_, with its relatively low dielectric constant, could be used to decrease the interface trap density, enhancing the stability and mobility of the device. Moreover, the crystallization of the SiO_2_ surface is smooth and uniform, resulting in improved device performance and more uniform films [51]. Devices incorporating the ZnO/SiO_2_/PMMA dielectric stack exhibit a reduced subthreshold swing and suppressed hysteresis, indicating a lower density of interfacial trap states. This behavior is consistent with previously reported effects of polymer passivation layers, with PMMA being known to mitigate interfacial trapping due to its relatively low dielectric constant. Accordingly, the observed improvement in device performance can be attributed to the combined passivation effect of PMMA and the stabilizing role of the SiO_2_/ZnO interface, as reported in the literature [52].

**Figure 5:**
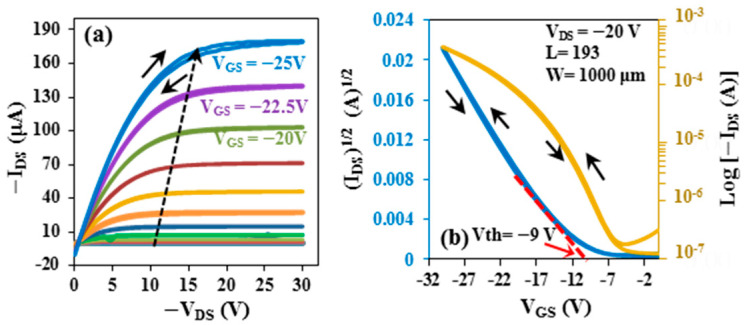
The (**a**) output and (**b**) transfer characteristics of OTFT on flexible PET substrate for the stable device.

To evaluate the stability of the fabricated OFET devices on flexible substrates, they were tested again in consecutive months after their first fabrication, all devices being vacuum-stored during intervening periods. The output and transfer characteristics of the flexible OFET devices were measured. The device’s stability against storage degradation, which was measured after one year of fabrication, is shown in Figure 5a,b. As can be seen from these figures, the flexible OFET devices exhibit typical p-type OFET behavior; they are still operating at high performance, with an on/off current ratio of 2.60 × 10^3^ and a high value of *μ*, the latter calculated to be 5.88 cm^2^/V.s with a *V_th_* of −9 V. Negligible hysteresis was also noticed for transfer characteristics as well as output. Moreover, a small leakage current and an absence of any significant drain offset current were observed in these devices.

Our reported OTFTs show significant performance improvements over time at high vacuum (about 10^−8^ mbar). Current hysteresis decreases, contact resistance may decrease by more than an order of magnitude, and field-effect mobility may roughly double. Instead of chemical doping, this enhancement results from thermally driven healing of structural point defects, especially shallow traps inside the polycrystalline grains [53,54,55]. The thin film can develop toward a more stable, crystalline structure even at ambient temperature because aging under vacuum promotes modest structural relaxation inside the pentacene layer. This reorganization enhances π–π stacking and decreases grain-boundary defects [56].

Table 3 summarizes the electrical parameters of OFET devices on glass and flexible substrates, with flexible OFET devices being measured both before and after bending and—in the case of stable devices—after one year of fabrication. The reported electrical parameters correspond to representative devices, while the statistical distribution of the electrical parameters obtained from 52 working devices is provided in the Supplementary Information.

As can be seen in Table 3, when we assessed the influence of substrate type on the device performance of two OFETs fabricated under identical processing conditions, one on a glass substrate and the other on a flexible substrate, the flexible-based OFET showed slightly higher field-effect mobility and a larger on/off ratio, while the glass-substrate device exhibited a modest increase in threshold voltage and reduced switching performance. These variations are primarily attributed to higher surface roughness, increased trap density at the dielectric/semiconductor interface, and possible differences in contact resistance associated with the glass substrate. Additionally, mechanical stress and the greater permeability of polymer substrates to moisture may contribute to the observed electrical shifts. Despite these differences, both devices maintained stable leakage currents, and the flexible OFET remained operational under bending, confirming its suitability for mechanically deformable electronics. Overall, the comparison shows that glass substrates cause only minor performance degradation, while flexible substrates offer significant advantages for flexible device applications.

Based on the results reported in the previous articles [57,58], bending the OFET device causes potential effects on two properties: the *I-V* characteristic, and electron mobility. The bending effect depends on whether the bending is downward or upward. In this work, the mobility and current–voltage characteristics of the OFET devices were measured in the downward bending direction, with slight differences in these measurements being exhibited. However, bending in the same direction led to a very small change in the OFET properties [59].

## 4. Conclusions

Fabrication and characterization of top-contact organic field-effect transistors (OFETs) on glass and flexible PET substrates based on dielectric layers of nano-crystalline ZnO, SiO_2_, and PMMA have been investigated in this study. We have successfully demonstrated high-performance flexible OFETs with high mobility of about 7.14 cm^2^/V.s. This mobility data is the best reported data among pentacene-based flexible OFETs in the literature. Good stability and a long lifetime were also observed in this work. This study highlights the superior performance of OFETs fabricated with a triple dielectric structure (ZnO/SiO_2_/PMMA) compared with previously reported devices using single or double dielectric layers. The harmony of the three layers of the insulating material, ZnO (30 nm), SiO_2_ (50 nm), and PMMA (70 nm), was investigated to enhance the good properties of the OFET device’s work on flexible substrates. There was negligible hysteresis in the output and transfer characteristics, and the stability of the OFET performance was confirmed after one year, when the behavior of the transfer characteristics was found to exhibit a slight change only. These findings confirm that the integration of multiple dielectric layers provides a synergistic effect, leading to enhanced charge transport and more stable device operation, making this approach highly promising for future high-performance and flexible OFET applications.

## Figures and Tables

**Table 1:** Electrical and mechanical properties of the flexible OFETs based on pentacene, compared with those obtained from devices previously reported in the literature.

V_th_ (V)	I_ON_/I_OFF_ Ratio	Mobility (cm^2^/V.s)	Substrate	Dielectric	Semiconductor	Publication Year
−1–−1.6	10^7^	0.5	PI	AlOx	Pentacene	2010 [24]
≈0	>10^3^	0.1–0.4	PEN	Mylar	TIPS-Pentacene	2012 [40]
−20.1	10^5^	1.51	PET	PVP/PMMA	Pentacene	2015 [35]
0.14	2.7 × 10^3^	0.14	PET	PS	TIPS-Pentacene	2022 [41]
≈1	≈10^5^	0.4	PI	PVP/PMMA	TIPS-Pentacene	2022 [42]
−5–−17	10^3^–10^6^	>1.0	PET, PEN, PI	PMMA, PVP, Al_2_O_3_, TiO_2_	Pentacene	2022 [43]
≈−2	>10^4^	0.3–0.5	PET	PMMA/SiO_2_	Pentacene + Carbon Dot–Ag	2025 [44]
−7	>10^3^	7.14	PET	ZnO/SiO_2_/PMMA	Pentacene	This work

**Table 2:** The effect of triple insulator layers on OFET devices based on pentacene.

Substrate	Dielectric	Dielectric Thickness (nm)	I_ON_/I_OFF_ Ratio	Mobility (cm^2^/V.s)	Threshold Voltage (V)	Channel Width/Length (µm)	References
Glass	PMMA	300	4.20 × 10^4^	1.32	−16.0	1000/95	[37]
Glass	PMMA	300	4.00 × 10^4^	0.27	−13.0	1000/140	[46]
Glass	PVP	350	2.66 × 10^2^	0.65	−7.0	2670/198	[46]
Glass	AlOx	60	-	4.0 × 10^−4^	−12.5	1000/95	[47]
Glass	AlOx/ODPA	120	-	0.016	−11.1	1000/95	[47]
Glass	AlOx/PMMA	110	-	0.10	−2.8	1000/95	[47]
Glass	ZnO/SiO_2_/PMMA	150	3.70 × 10^3^	6.65	−9.0	1000/193	This work
Flexible	ZnO/SiO_2_/PMMA	150	7.10 × 10^3^	7.14	−7.0	1000/193	This work

**Table 3:** The electrical parameters of OFET devices on glass and flexible substrates.

Structure	*V_th_* (V)	*I_ON_/I_OFF_* Ratio	Mobility (cm^2^/V.s)
On glass substrate	−9	3.70 × 10^3^	6.75
On flexible substrate before bending	−7	7.10 × 10^3^	7.14
On flexible substrate after bending	−9	4.40 × 10^3^	6.88
Stable devices	−9	2.60 × 10^3^	5.88

## Data Availability

No new data were created.

## Supplementary Materials

The following supporting information can be downloaded at: https://www.mdpi.com/article/10.3390/mi17030382/s1. Section S1. Device Fabrication Yield and Reproducibility. Section S2. Statistical Analysis Before Bending (n = 52). Section S3. Statistical Analysis After Bending (All Devices, n = 52). Section S4. High-Performance Device Stability After Bending (n = 48). Section S5. Discussion.
