# Supplementary material for: Nanoscale Thin-Film Flexible Organic Field-Effect Transistors with Triple PMMA/SiO2/ZnO Gate Insulator Layers
Source: Micromachines (Basel). 2026 Mar 21;17(3):382. doi: 10.3390/mi17030382 (PMC13028382; doi:10.3390/mi17030382)
Supplement: Supplementary file 1 [file micromachines-17-00382-s001.zip › micromachines-3954427-supplementary.pdf]

Supplementary Information

Statistical Analysis and Mechanical Robustness of Flexible OFET Devices

S1. Device Fabrication Yield and Reproducibility

A total of 60 flexible OFET devices were fabricated under identical processing conditions. Among these, 52 devices (86.7%) exhibited high-performance electrical characteristics prior to mechanical deformation. Following cyclic bending tests, 48 devices retained high-performance operation, corresponding to 80% of the total fabricated devices and 92.3% retention relative to the initially high-performing population. These results demonstrate a high fabrication yield and excellent reproducibility of the device architecture.

S2. Statistical Analysis Before Bending (n = 52)

The key electrical parameters were statistically analyzed to evaluate device uniformity. Mean values and standard deviations (SD) were extracted from 52 working devices.

Parameter	Mean ± SD
Threshold Voltage (Vth)	-9.44 ± 2.36 V
Field-Effect Mobility	6.24 ± 0.93 $cm^2/V·s$
On/Off Current Ratio	$(4.64 ± 1.96) × 10^3$

S3. Statistical Analysis After Bending (All Devices, n = 52)

After mechanical bending, the statistical distribution of the electrical parameters was re-evaluated to assess strain-induced effects on device performance.

Parameter	Mean ± SD
Threshold Voltage (Vth)	-11.19 ± 2.63 V
Field-Effect Mobility	5.23 ± 1.73 $cm^2/V·s$
On/Off Current Ratio	$(4.69 ± 2.02) × 10^3$

S4. High-Performance Device Stability After Bending (n = 48)

To further evaluate mechanical robustness, only the 48 devices that retained high-performance characteristics after bending were statistically analyzed. The extracted mobility was 5.59 ± 0.79 $cm^2/V·s$, corresponding to approximately 89.6% mobility retention compared to the pre-bending average. The relatively narrow standard deviation confirms that the majority of devices maintain stable charge transport under mechanical stress.

S5. Discussion

A moderate negative shift in threshold voltage (~1.75 V) was observed after bending, which can be attributed to strain-induced charge trapping at the semiconductor/dielectric interface or localized interface state generation. Despite this shift, the field-effect mobility and on/off current ratio remained statistically stable within experimental variation limits. The preservation of high mobility retention (~89.6%) and the high percentage of operational devices (92.3% of initially high-performing devices) collectively confirm the mechanical durability and structural integrity of the flexible OFET platform. Overall, the narrow statistical distributions and high device yield demonstrate excellent reproducibility and robustness, meeting the reliability standards expected for advanced flexible electronic applications.
